# Case Report: Paralytic rabies masquerading as traumatic spinal cord injury: a diagnostic pitfall in trauma surgery

**DOI:** 10.3389/fmed.2026.1954968

**Published:** 2026-10-13

**Authors:** Rui Zhao, Lian-Jing Liang, Si-Fen Lu, Rong Yao, Tao Cheng

**Affiliations:** 1 Department of Emergency Medicine, Institute of Disaster Medicine and Institute of Emergency Medicine, West China Hospital, Sichuan University, Chengdu, Sichuan, China; 2 West China School of Nursing, Sichuan University, Chengdu, China; 3 Precision Medicine Key Laboratory of Sichuan Province and Precision Medicine Center, West China Hospital, Sichuan University, Chengdu, China

**Keywords:** cognitive bias, diagnostic error, metagenomic sequencing, paralytic rabies, rabies, spinal cord injury

## Abstract

Rabies is a uniformly fatal zoonotic encephalitis. Its paralytic form, which accounts for approximately 20% of cases, frequently resembles acute spinal cord injury (SCI). When there is a coincident history of trauma, diagnostic anchoring may wrongly attribute neurological decline to the injury, thereby delaying the correct diagnosis. We describe the case of a 26-year-old man who developed progressive, ascending paralysis of the lower limbs 10 days after a 3-m fall. At a secondary hospital, he was clinically diagnosed with traumatic SCI without spinal MRI. On transfer, reassessment revealed a fox bite 4 months earlier and features of the hydrophobia variant (a sensation of water “entering the brain” during swallowing). Cerebrospinal fluid metagenomic next-generation sequencing (mNGS) detected Rabies lyssavirus within 48 h, showing 100% sequence identity to Chinese isolates spanning the N and L genes. This finding was subsequently confirmed by the Sichuan Provincial Center for Disease Control and Prevention (CDC) using standard reference testing on peripheral blood. The patient subsequently died of autonomic and respiratory failure. This case highlights how recent trauma can obscure a fatal infectious cause. A clinical diagnosis of SCI made without neuroimaging should be regarded as provisional, and mNGS can provide an unbiased, rapid means of resolving diagnostically challenging neurological presentations.

## 1 Introduction

Rabies is an almost invariably fatal viral disease. In China, after 16 consecutive years of decline, human rabies cases increased by 36.9% in 2024, with 167 reported cases (1). The “furious” form is readily recognized by hydrophobia and agitation, whereas the “paralytic” form causes progressive flaccid paralysis and is frequently mistaken for Guillain–Barré syndrome (GBS) or acute traumatic spinal cord injury (SCI) (2, 3). In trauma practice, a coincident fall or impact can bias clinicians toward structural explanations, creating a diagnostic blind spot for non-traumatic causes (4). We describe a case of paralytic rabies initially misdiagnosed as traumatic SCI after a confounding fall and illustrate how metagenomic next-generation sequencing (mNGS) resolved the diagnostic uncertainty.

## 2 Case presentation

### 2.1 Initial presentation and history

A 26-year-old previously healthy man was referred to our emergency department with a 10-day history of progressive lower-limb paralysis. He had no relevant family history. Twenty days before the onset of paralysis, he fell 3 m while working. During the days immediately following the fall, he had been ambulatory with only mild back pain and no neurological deficit. Because of the temporal association with the fall, a secondary hospital diagnosed traumatic spinal cord injury; no spinal MRI was performed and he received conservative treatment for a presumed spinal concussion.

Ten days after the fall, he developed progressive ascending weakness, starting in the distal lower limbs and extending proximally, accompanied by ascending sensory loss with a clearly defined sensory level at the T10 dermatome. Examination of deep tendon reflexes revealed generalized areflexia in the lower limbs (0/4) and diminished reflexes in the upper limbs (1/4). Plantar responses were bilaterally extensor. Cranial nerve examination revealed bilateral abducens nerve palsy (limitation of lateral gaze) and a depressed gag reflex. Bowel and bladder involvement was manifest as urinary retention and constipation, requiring catheterization and manual evacuation.

Three days before the onset of complete paralysis (i.e., 7 days after the fall, and 3 days before admission), his family reported that he described a sensation of water “entering the brain” when swallowing—a unique subjective complaint that we interpreted as reflecting pharyngeal spasm or brainstem/bulbar sensory dysesthesia (Figure 1).

**Figure 1:**
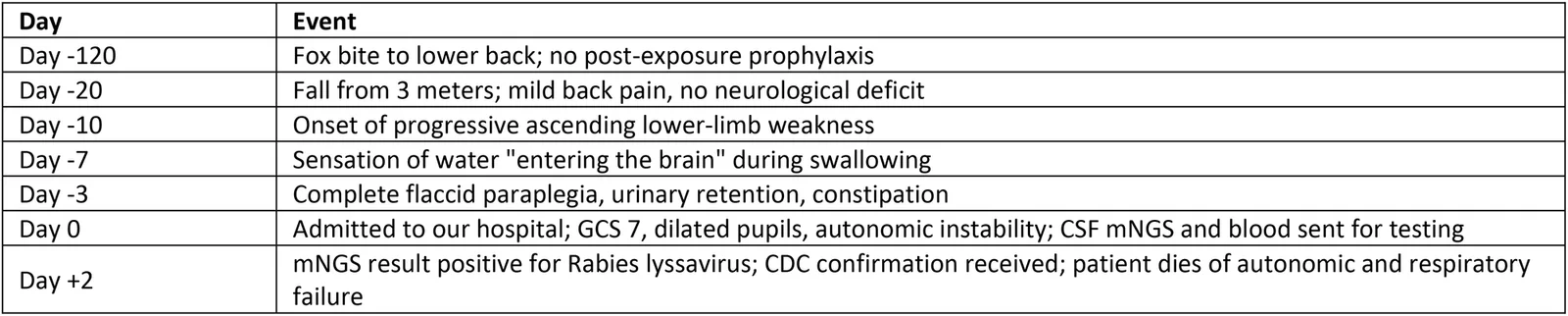
Timeline of the episode of care (following CARE guidelines).

### 2.2 Clinical examination and differential diagnosis

On arrival at our institution, his condition had deteriorated to coma (Glasgow Coma Scale (GCS) score 7) with fixed, dilated pupils (7 mm, bilateral, sluggishly reactive). Examination showed complete flaccid paraplegia (muscle strength 0/5) with a sensory level at T10, loss of proprioception and vibration sense below the knees, and autonomic instability, including refractory bradycardia (heart rate 40–50 bpm) and hypotension (systolic blood pressure 80–90 mmHg). The pattern of findings—asymmetric cranial nerve involvement (bilateral abducens palsy, depressed gag), areflexic paraplegia, ascending sensory level, and early autonomic dysfunction—indicated a brainstem and spinal cord process, rather than a focal traumatic myelopathy.

Critical reevaluation disclosed a fox bite to the lower back 4 months earlier, for which he had not received postexposure prophylaxis (PEP). The bite wound on the lower back had healed without local signs of inflammation or infection.

Neuroimaging and Laboratory Workup: Head CT performed on admission was unremarkable. No spinal MRI was performed at either the referring hospital or our institution, as the patient's rapid neurological deterioration (declining GCS and hemodynamic instability) precluded safe transport to the MRI suite. This absence of neuroimaging meant that the diagnosis of SCI remained purely provisional and unconfirmed radiologically.

Cerebrospinal fluid (CSF) analysis revealed 12 leukocytes/μL (reference <5 leukocytes/μL) and protein 0.85 g/L (reference 0.15–0.45 g/L). The CSF/blood glucose ratio was 0.62 (CSF glucose 4.2 mmol/L, serum glucose 6.8 mmol/L). Serum creatine kinase was not elevated, and no electrophysiological studies (nerve conduction studies or electromyography) were performed due to the patient's critical condition—acknowledged as a limitation.

To systematically evaluate the differential diagnosis of acute flaccid paralysis, we constructed Table 1.

**Table 1:** Differential diagnosis of acute flaccid paralysis in this patient.

Diagnosis	Features supporting	Features against	Key diagnostic tests to rule in/rule out
Guillain–Barré syndrome (GBS)	Ascending flaccid paralysis, areflexia	Absence of albuminocytologic dissociation (CSF protein 0.85 g/L—elevated but modest); rapid progression to brainstem involvement; hydrophobia; protracted prodrome (4 months) is highly atypical	Nerve conduction studies/electromyography (EMG), CSF albuminocytologic dissociation, antiganglioside antibodies
Acute transverse myelitis	Sensory level, paraplegia, sphincter dysfunction	Lack of spinal MRI confirmation; brainstem and cranial nerve involvement (abducens palsy, gag reflex depression) extend beyond the spinal cord	Spinal MRI, CSF oligoclonal bands, IgG index, aquaporin-4 antibodies
Spinal cord infarction	Acute onset of paraplegia, sensory level, sphincter loss	No vascular risk factors; progressive ascending course over 10 days (rather than abrupt onset); CSF pleocytosis; hydrophobia variant	Spinal MRI (diffusion-weighted imaging), vascular imaging
Compressive myelopathy	Progressive paraplegia, sensory level, sphincter dysfunction	No spinal MRI; lack of radicular pain; absence of a spinal mass or fracture on clinical grounds; brainstem involvement	Spinal MRI or CT myelography
Paralytic rabies	Hydrophobia variant; history of fox bite with no PEP; CSF mNGS confirmation; CDC confirmation	—	CSF mNGS, rabies PCR/DFA on saliva or skin biopsy, rabies serology (serum/CSF antibodies)

a In this patient, GBS was deemed less likely primarily due to early urinary retention, extensor plantar responses, and rapid progression to fatal encephalopathy, along with the absence of typical albuminocytologic dissociation. The diagnosis of rabies was ultimately confirmed by CSF mNGS and validated by CDC reference testing.

### 2.3 Laboratory and mNGS findings

CSF mNGS (Illumina platform) identified low-abundance sequence reads specific to Rabies lyssavirus within 48 h. The sequencing metrics are summarized in Table 2 and Figure 2. Two unique reads (43 bp each) were aligned to the Rabies lyssavirus reference genome (NC_001542), covering 0.721% of the viral genome. These reads mapped to the N gene (positions 537–579) and L gene (positions 11,650–11,692), with 100% identity to multiple Chinese isolates (e.g., KY982923.1, KY175229.1). No reads were detected in the no-template control, excluding cross-contamination. Concurrently, peripheral blood samples sent to the Sichuan Provincial Center for Disease Control and Prevention (CDC) were positive for Rabies lyssavirus by standard reference testing, officially confirming the diagnosis.

**Table 2:** mNGS detection metrics for rabies lyssavirus.

Metric	Result
Specific reads for Rabies lyssavirus	2 (43 bp each)
Genome coverage	0.721%
Target genes	N (537–579), L (11,650–11,692)
Identity	100% (to multiple Chinese isolates)
Negative control	0 reads

**Figure 2:**
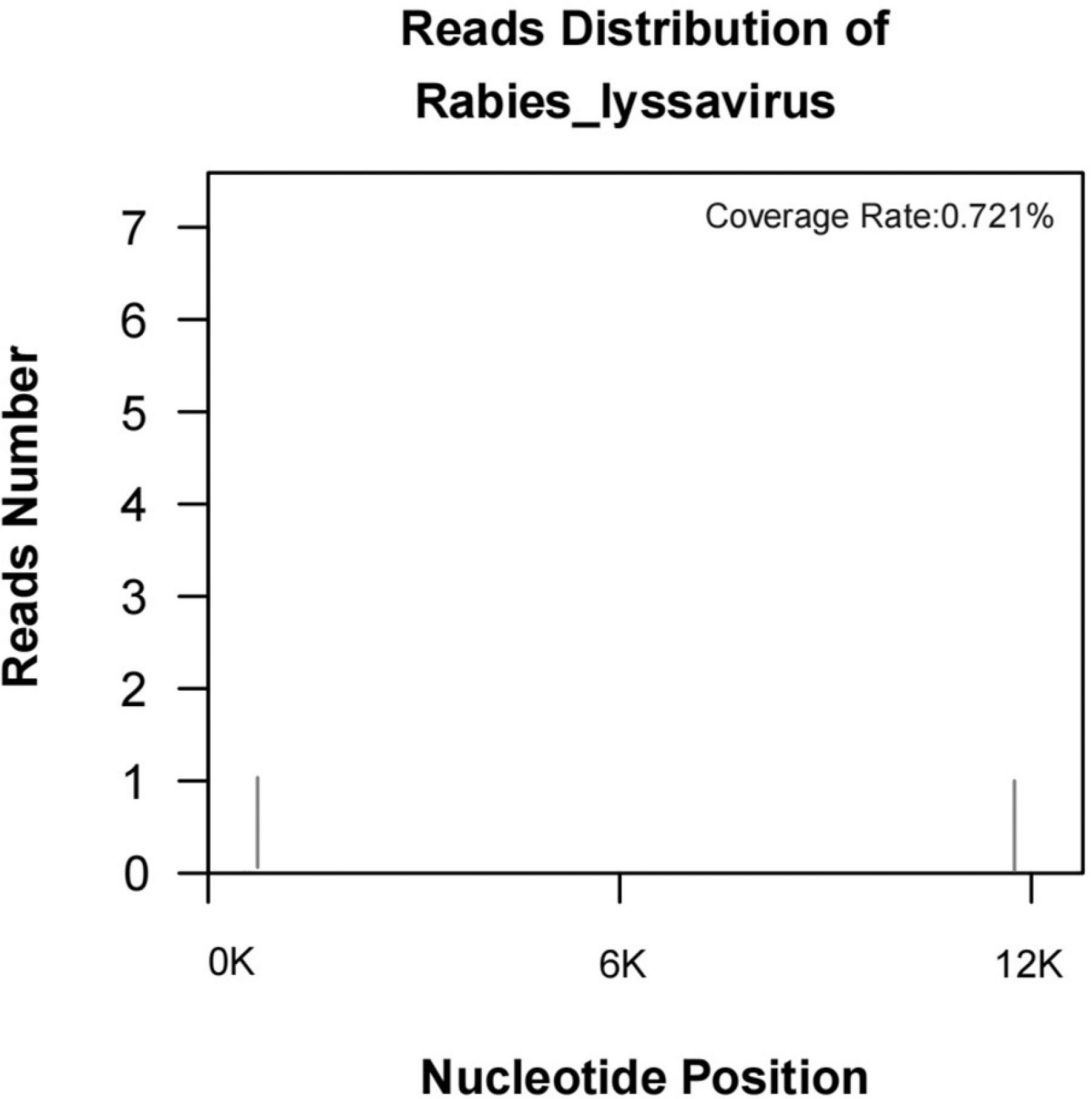
Read coverage of the rabies virus genome. Metagenomic sequencing reads from patient cerebrospinal fluid aligned to the rabies virus reference genome (NC_001542). Mapping of specific reads to distinct regions of the N and L genes confirmed the presence of Rabies lyssavirus genomic material.

## 3 Discussion

Paralytic rabies manifests as ascending paralysis due to viral spread and inflammatory degeneration of the spinal cord and brainstem (2, 5). Notably, a case series from India reported that among 83 patients initially treated for Guillain–Barré syndrome, three (3.6%) were ultimately diagnosed with paralytic rabies (6). This case illustrates anchoring bias, a cognitive error in which clinicians overrely on an initial datum (the traumatic event) to account for all subsequent findings (4).

Cognitive debiasing strategies are essential in trauma practice. Structured diagnostic time-outs, cognitive checklists, and multidisciplinary team reviews can help counteract the tendency to prematurely close a diagnosis. In this case, a rigorous neurological localization exercise—recognizing that the combination of brainstem signs (abducens palsy, depressed gag), spinal sensory level, and autonomic instability could not be explained by a single mechanical impact—would have prompted earlier consideration of infectious etiologies.

Public health implications are noteworthy. The resurgence of rabies in China, with a 36.9% increase in 2024, underscores the need for heightened public awareness regarding timely PEP, even for seemingly minor animal exposures. Our patient had no visible wound inflammation at the time of admission, which may have contributed to the oversight. Health education campaigns should emphasize that any animal bite—especially from wildlife like foxes—warrants immediate medical evaluation and PEP.

Comparison of mNGS with traditional assays: Standard direct fluorescent antibody (DFA) testing and RT-PCR are highly specific but require targeted primers and are often less sensitive in early or atypical cases. In contrast, mNGS offers unbiased, broad-spectrum pathogen detection with a turnaround time of 24–48 h, making it invaluable in diagnostically challenging scenarios. A 2025 systematic review confirmed that conventional assays have suboptimal sensitivity, particularly in early-stage infection, further supporting the use of mNGS in such cases (7).

The role of MRI in this case deserves careful consideration. While emergent spinal MRI would not have directly diagnosed rabies, it would have been essential to rule out compressive traumatic myelopathy. Its absence left the diagnosis of SCI unconfirmed, and the clinical picture—with ascending paralysis, bulbar involvement, and hydrophobia—should have prompted immediate search for non-traumatic causes. We therefore suggest that in similar cases, although MRI may be logistically challenging in unstable patients, every effort should be made to obtain spinal imaging to exclude surgically treatable lesions, while simultaneously pursuing infectious workup.

We acknowledge several limitations: the lack of spinal MRI and electrophysiological studies, and the absence of additional CSF biomarkers such as lactate, oligoclonal bands, and IgG index. These are now stated explicitly.

## 4 Conclusions

This case highlights four critical takeaways for clinicians and trauma surgeons:

• A clinical diagnosis of spinal cord injury made without confirmatory neuroimaging must remain strictly provisional.

• Ascending flaccid paralysis accompanied by bulbar signs or autonomic instability should immediately prompt investigation for non-traumatic myelopathy or encephalitis.

• Atypical variants of hydrophobia (such as abnormal sensation during swallowing) are important clinical clues pointing toward rabies.

• Unbiased mNGS should be deployed early in cases of progressive, diagnostically uncertain neurological deficits.

## Data Availability

The raw data supporting the conclusions of this article will be made available by the authors, without undue reservation.
